# How to move an amphipathic molecule across a lipid bilayer: different mechanisms for different ABC transporters?

**DOI:** 10.1042/BST20160040

**Published:** 2016-06-09

**Authors:** Frederica L. Theodoulou, David J. Carrier, Theresia A. Schaedler, Stephen A. Baldwin, Alison Baker

**Affiliations:** * Biological Chemistry and Crop Protection Department, Rothamsted Research, Harpenden, AL5 2JQ, U.K.; † Centre for Plant Sciences, School of Molecular and Cellular Biology, University of Leeds, Leeds LS2 9JT, U.K.; ‡ Astbury Centre for Structural Molecular Biology, University of Leeds, Leeds LS2 9JT, U.K.

**Keywords:** ABC transporter, acyl-CoA, asymmetry, mechanism, β-oxidation, peroxisome, thioesterase

## Abstract

Import of β-oxidation substrates into peroxisomes is mediated by ATP binding cassette (ABC) transporters belonging to subfamily D. In order to enter the β-oxidation pathway, fatty acids are activated by conversion to fatty acyl-CoA esters, a reaction which is catalysed by acyl-CoA synthetases (ACSs). Here, we present evidence for an unusual transport mechanism, in which fatty acyl-CoA substrates are accepted by ABC subclass D protein (ABCD) transporters, cleaved by the transporters during transit across the lipid bilayer to release CoA, and ultimately re-esterified in the peroxisome lumen by ACSs which interact with the transporter. We propose that this solves the biophysical problem of moving an amphipathic molecule across the peroxisomal membrane, since the intrinsic thioesterase activity of the transporter permits separate membrane translocation pathways for the hydrophobic fatty acid moiety and the polar CoA moiety. The cleavage/re-esterification mechanism also has the potential to control entry of disparate substrates into the β-oxidation pathway when coupled with distinct peroxisomal ACSs. A different solution to the movement of amphipathic molecules across a lipid bilayer is deployed by the bacterial lipid-linked oligosaccharide (LLO) flippase, PglK, in which the hydrophilic head group and the hydrophobic polyprenyl tail of the substrate are proposed to have distinct translocation pathways but are not chemically separated during transport. We discuss a speculative alternating access model for ABCD proteins based on the mammalian ABC transporter associated with antigen processing (TAP) and compare it to the novel mechanism suggested by the recent PglK crystal structures and biochemical data.

## Fatty acid import into peroxisomes: the role of ABC transporters

A distinctive function of peroxisomes is the β-oxidation pathway in which fatty acids (FA) are degraded with the concomitant generation of acetyl-CoA [1]. Genetic and biochemical studies have shown that import of substrates into peroxisomes for β-oxidation is mediated by ATP binding cassette (ABC) proteins belonging to subfamily D [2–5]. ABC transporters have a core structure consisting of two nucleotide binding domains (NBDs) and two transmembrane domains (TMDs). The NBDs form a ‘sandwich dimer’ capable of binding and hydrolysing ATP, whereas the TMDs, each composed of multiple α-helices, bind and translocate substrates [6]. In animals and fungi, peroxisomal ABCD (ABC subclass D protein) proteins are dimers of two TMD-NBD ‘half transporters’, whereas in plants the equivalent transporters are expressed from a single open reading frame which encodes a protein with the topology, TMD-NBD-TMD-NBD.

**Figure 1:**
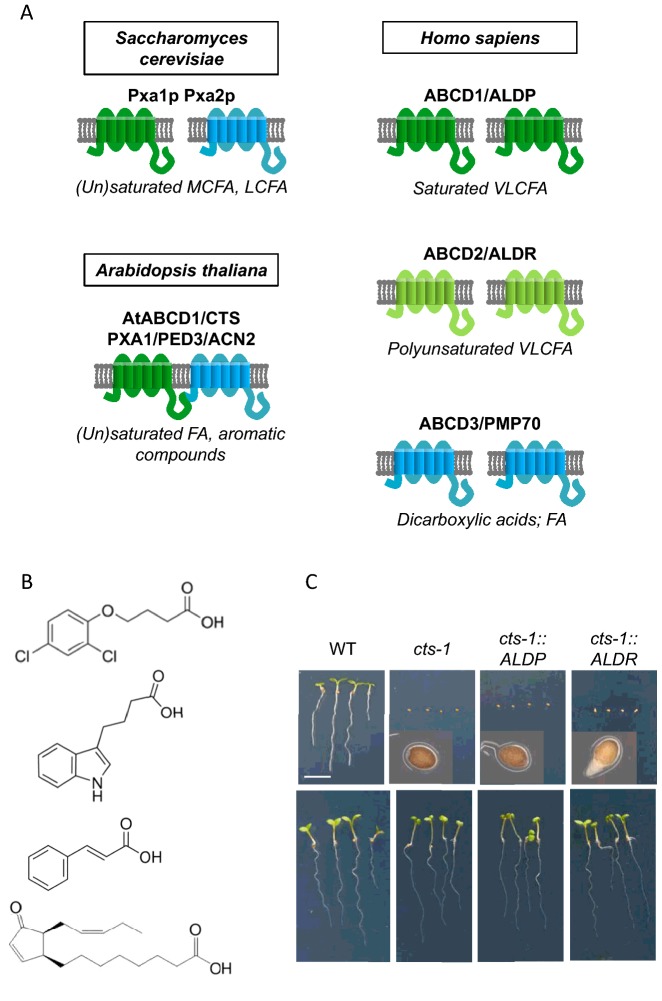
Domain organization and substrate specificity of peroxisomal ABC transporters (**A**) Cartoons showing domain organization of yeast, human and plant peroxisomal ABCD proteins. Preferred substrates are indicated below each transporter. (**B**) Non-fatty acid compounds which are imported by CTS. Free acids are shown for simplicity but note that the ABCD transporters are thought to accept CoA esters. From top to bottom: 2,4-dichlorobutric acid, IBA, *trans*-cinnamic acid, OPDA. (**C**) Human ALDR but not ALDP complements the Arabidopsis *cts-1* mutant for seed germination, indicating transport of OPDA. Images were recorded after 5 days on medium containing sucrose (lower panel) or without sucrose (upper panel). In the lower panel, seeds were induced to germinate by mechanical disruption of the seed coat. Scale bar 5 mm; insets are magnified 10×. This composite image was originally published in [38]: Zhang, X., De Marcos Lousa, C., Schutte-Lensink, N., Ofman, R., Wanders, R.J., Baldwin, S.A., Baker, A., Kemp, S. and Theodoulou, F.L. (2011) Conservation of targeting but divergence in function and quality control of peroxisomal ABC transporters: an analysis using cross-kingdom expression. Biochem. J. **436**, 547–557 http://www.biochemj.org/content/436/3/547.long.

Baker's yeast has a single heterodimeric peroxisomal ABC transporter, Pxa1p/Pxa2p (Figure 1A), required for utilization of fatty acids [7,8] and the *pxa1/2Δ* mutant has proved to be a useful tool for expression and analysis of ABCD proteins from other organisms [9–12]. Although Pxa1/2p has been proposed to transport acyl-CoAs of different chain lengths [8,13], medium chain free (non-esterified) fatty acids are also imported into yeast peroxisomes via a parallel, ABC-independent route which requires the peroxisomal acyl-CoA synthetase (ACS), Faa2p and the adenine nucleotide translocator, Ant1p [8,14,15].

Human peroxisomes harbour three ABC transporters: adrenoleukodystrophy protein, ALDP/ABCD1, adrenoleukodystrophy related protein, ALDR/ABCD2 and the 70 kDa peroxisomal membrane protein, PMP70/ABCD3 (Figure 1A) [2,3]. A fourth member of human subfamily D, ABCD4, is localized to lysosomes and required for cobalamin export to the cytosol [16]. The three peroxisomal transporters display overlapping but distinct substrate specificities [2,3,5]. Expression in the yeast *pxa1/2Δ* mutant coupled with β-oxidation measurements indicates that ALDP preferentially transports saturated very long chain fatty acids (VLCFA) C_24:0_ and C_26:0_; ALDR has a preference for VLC unsaturated FA such as C_22:6_ but can also transport C_22:0_ and PMP70 accepts a range of long chain unsaturated, branched and dicarboxylic acids [10–12]. These results are largely supported by cross-complementation experiments and lipid analysis of tissues and cells in which the individual transporters have been mutated or knocked down [17–19]. Until recently, only ALDP was associated with human disease, its dysfunction or absence resulting in X-linked adrenoleukodystrophy, an inherited metabolic storage disorder characterized by accumulation of VLCFA in tissues [20]. However, PMP70 has recently been associated with a defect in bile acid synthesis [21].

As human ABCs are encoded as half-size transporters, it has been suggested that they could potentially heterodimerize to generate additional transport functions, by analogy to *Drosophila* eye pigment transporters belonging to subfamily G. Although it is clear that human ABCD proteins can indeed heterodimerize when co-expressed and heterodimers have occasionally been detected *in vivo* [22–24] several lines of evidence indicate that they are predominantly homodimeric *in vivo* [2,10–12,25].

The model plant, *Arabidopsis thaliana* has a single peroxisomal ABC transporter, AtABCD1 (also known as CTS, PXA1, PED3 and ACN2, and referred to hereafter as CTS; Figure 1A) [4,26–29]. Phylogenetic analysis reveals that the fusion of two half transporters into a single pseudoheterodimeric protein was a single event in the evolution of the green plant lineage which occurred before the divergence of bryophytes [30], however in monocotylendonous plants (which include cereal crops such as barley and wheat), the *ABCD1* gene has been duplicated and appears to be undergoing functional specialization [31]. A second *Arabidopsis* member of the subfamily D, AtABCD2 is localized to plastids and apparently does not play a role in β-oxidation [32,33].

In addition to a major role in lipid metabolism, plant peroxisomes process numerous secondary compounds and hormones by β-oxidation [1]. Several studies have shown that AtABCD1/CTS is the entry point for the diverse substrates of the pathway, apparently exhibiting a broad substrate specificity which includes not only various fatty acids and acetate but also ring-containing compounds such as the jasmonic acid precursor, 12-oxophytodienoic acid (OPDA) and synthetic and natural auxins, 2,4-dichlorophenoxybutryric acid and indole butyric acid (IBA) (Figure 1B) (reviewed in [4,34,35]). A role for CTS in the synthesis of benzoylated metabolites has also been discovered recently [36,37]. The different phenotypes of the *cts-1* null mutant provide a test-bed to assess the substrate specificity of heterologous ABCD proteins. Although human ALDP and ALDR were targeted correctly to the peroxisome in plants, only ALDR was able to complement the germination phenotype of *cts-1*, consistent with the ability to import OPDA but neither transporter could restore seed oil mobilization or IBA metabolism, in line with their narrower substrate specificity [38] (Figure 1C).

## Transport substrates and mechanism

In order to enter β-oxidation, fatty acids must be activated by thioesterification to Coenzyme A, an ATP-requiring reaction which is a catalysed by ACSs belonging to the family of acyl activating enzymes (AAEs). AAEs are present in both the cytosol and the peroxisome [8,39], therefore in principle, CoA esters could be formed either outside or inside the peroxisome. There has been much debate regarding the identity of the molecular species transported by ABCD proteins, specifically whether the transporters accept CoA esters or free acids. Experiments in which the yeast plasma membrane was selectively permeabilized suggest that Pxa1p/2p accepts long chain fatty acyl-CoAs rather than free fatty acids [13]. In agreement with this, glycosomes (specialized peroxisomes) from *Trypanosoma brucei* incorporate radiolabelled oleoyl-CoA in an ABC transporter-dependent manner [40] and isolated human peroxisomes are able to oxidize C_26:0_-CoA [19]. Stimulation of CTS-dependent ATPase activity by fatty acyl-CoAs together with their accumulation in *Arabidopsis cts* null mutants and the ability of CTS to complement the yeast *pxa1/2Δ* mutant [9,27] suggested that it is also a transporter of CoA esters. However, the requirement of peroxisomal ACSs and adenine nucleotide translocator activity for lipid degradation [41–43] pointed to free acids as the transported substrates for CTS. In an attempt to resolve this paradox, a model was suggested in which the transporter accepts FA-CoA at the cytosolic side of the membrane and CoA is cleaved from the fatty acyl-CoA substrate during or after transport, requiring re-activation by ATP-dependent peroxisomal ACS in the lumen [41]. The first direct evidence in support of this ABCD transporter mechanism was obtained by isotopic labelling in yeast, which revealed that CoA is indeed liberated during fatty acid transport into yeast peroxisomes but did not identify the source of the thioesterase activity or the compartment in which the CoA is released [44]. A requirement for peroxisomal ACS activity in FA β-oxidation was demonstrated for complementation of *pxa1/2Δ* by ALDR, ALDP and CTS [44,45] and the *Arabidopsis* peroxisomal long chain ACSs, LACS6 and 7 were shown to reside with the transporter in a high molecular mass complex *in planta* [45]. It was subsequently discovered that insect cell membranes expressing CTS exhibited ATP-dependent acyl-CoA thioesterase activity. Although these measurements were not performed on purified, recombinant protein, a mutation in CTS which ablates fatty acid β-oxidation *in planta* [27] (and also when CTS is expressed in yeast) markedly reduced thioesterase activity of CTS when expressed in insect cells but had less effect on the basal ATPase activity. Taken together, these findings suggest that the activity is a property of the transporter and not provided by an associated heterologous protein [45].

## How do ABCD transporters bind and cleave CoA esters?

**Figure 2:**
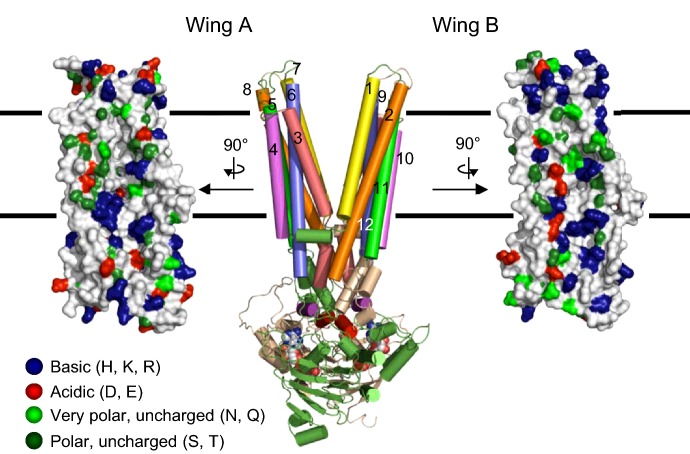
A putative permeant binding cavity identified by homology modelling of CTS/AtABCD1 Outward (lumen)-facing conformation of CTS, modelled on Sav1866 2HYD structure [46,47]. The transmembrane helices form two ‘wings’, designated **A** and **B**, which surround a central putative permeant binding cavity. α-Helices are indicated by numbers and colour-coded. The inner surfaces of the cavity are shown in space-filling representation and colour-coded according to charge.

At present, no structural data are available for ABCD proteins but homology modelling has offered insights into their architecture. Modelling CTS on the ‘gold standard’ Sav1866 2HYD structure of Dawson and Locher [46] suggests that the coupling helices contained within the long intracellular loops linking TM helices interact with the NBD of the opposite subunit in an arrangement termed ‘domain swapping’ [47]. Interestingly, ABCD proteins have a significant number of conserved hydrophilic residues in predicted transmembrane helices, several of which are predicted to face into a putative permeant binding cavity identified in Sav1866 (Figure 2) [46]. These residues are potential candidates for a CoA binding site. The protein structure database contains over 200 structures with bound CoAs and it is evident that different enzymes bind CoAs in different conformations, with backbone and side-chain hydrogen bonds and salt bridges being involved to variable extents. Given that 2HYD represents an outward facing conformation, relating in this case to a peroxisome lumen-facing state, this would correspond to a low affinity binding site in an alternating access model. The lumen-facing model of CTS features a putative permeant binding cavity with a polar surface which is large enough to accommodate an acyl CoA head group (Figure 2).

The structural basis for intrinsic acyl-CoA thioesterase activity of ABCD transporters is currently unclear, since these proteins do not exhibit sequence similarity to soluble thioesterases. However, thioesterases are a diverse class of enzymes which fall into 23 different families, with different folds and mechanisms and little primary sequence identity [48]. Homology modelling predicts that ABCD proteins do not have novel accessory domains in which the thioesterase activity might reside, implying that the thioesterase catalytic site must have evolved in the context of the core ABC transporter domains and the charged residues in TM helices may be important in this respect. It is also notable that soluble thioesterases are not known to require or be stimulated by ATP, unlike the activity exhibited by CTS. An interesting topic for future research will be to explore the structural and functional relationship between thioesterase activity, ATP binding, hydrolysis and substrate translocation in ABCD proteins. The contribution of peroxisomal AAEs with different substrate specificities to channelling of diverse substrates into β-oxidation is also worthy of exploration.

## Towards an understanding of the transport mechanism: insights from lipid-linked oligosaccharide flippase PglK

**Figure 3:**
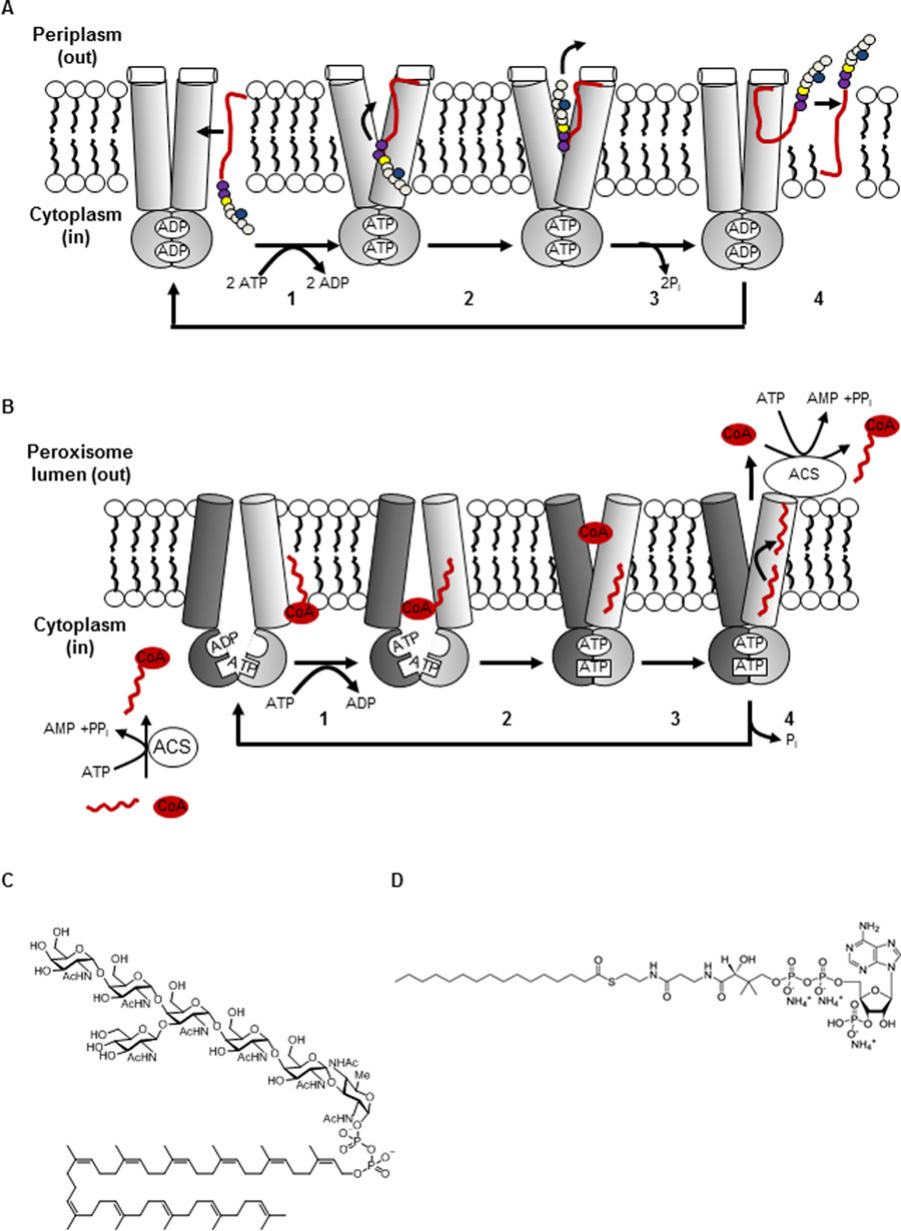
Proposed transport mechanisms for PglK and AtABCD1 (**A**) PglK flipping mechanism for LLO (redrawn after [50]: Perez, C., Gerber, S., Boilevin, J., Bucher, M., Darbre, T., Aebi, M., Reymond, J.-L. and Locher, K.P. (2015) Structure and mechanism of an active lipid-linked oligosaccharide flippase. Nature **524**, 433–438). The cycle starts with ADP-bound transporter in the outward occluded conformation; LLO approaches the transporter from the membrane and LLO polyprenyl tail (red) interacts with external helix (white cylinder) (1) ATP/ADP exchange; transporter adopts open-outward conformation. (2) Pyrophosphate-oligosaccharide head group (coloured circles) of LLO enters outward-facing cavity. (3) ATP hydrolysis and return to outward occluded conformation. (4) Release of LLO head group and polyprenyl tail, accomplishing flipping in the membrane. The transporter is now ready to undergo another cycle (indicated by arrow). (**B**) ABCD alternating access/thioesterase mechanism (modified from [55]: Procko, E., O'Mara, M.L., Bennett, W.F.D., Tieleman, D.P. and Gaudet, R. (2009) The mechanism of ABC transporters: general lessons from structural and functional studies of an antigenic peptide transporter. FASEB J. **23**, 1287–1302 and [4]: Theodoulou, F.L., Baldwin, S.A., Baldwin, J.M. and Baker, A. (2014) Plant peroxisomal ABC transporters: flexible and unusual. In Plant ABC Transporters, Signaling and Communication in Plants (Geisler, M., ed.), vol. 22, Chapter 16, Springer-Verlag, Berlin, Heidelberg). Asymmetric ABCD proteins have one canonical composite nucleotide binding site (oval) and one degenerate site (rectangle). The acyl-CoA substrate (red) is formed outside peroxisomes by the action of cytosolic or microsomal ACSs. The transport cycle starts in the open-inward conformation, in which at least one nucleotide binding site is occupied by ADP. (1) The hydrophobic moiety of the acyl-CoA substrate partitions into the membrane where it interacts with the TMDs; the CoA moiety binds to a hydrophilic site. Substrate binding stimulates ATP–ADP exchange. (2) ATP-dependent NBD closure switches the transporter to the outward-facing conformation and favours thioester cleavage. CoA moiety enters outward-facing cavity. (3) Fatty acid flip-flop in the membrane, CoA release to the peroxisome lumen and ACS-dependent re-esterification. (4) ATP hydrolysis weakens contacts between NBDs and NBD opening restores inward-facing conformation. In symmetrical ABCD proteins such as ALDP, both nucleotide binding sites are capable of hydrolysing ATP. (**C**) Structure of the LLO GlcGalNAc_5_Bac-PP-undecaprenyl (a substrate of PglK). Reproduced with permission from [50]: Perez, C., Gerber, S., Boilevin, J., Bucher, M., Darbre, T., Aebi, M., Reymond, J.-L. and Locher, K.P. (2015) Structure and mechanism of an active lipid-linked oligosaccharide flippase. Nature **524**, 433–438. (**D**) Structure of 16:0-CoA (a substrate of CTS and other ABCD transporters).

Several ABC transporters mediate the transport of large, amphipathic molecules across membranes, posing intriguing questions regarding the nature of the substrate binding sites and translocation pathway for such substrates. The thioesterase activity of CTS may circumvent the requirement for a binding site which accommodates amphipathic molecules, since it allows for the possibility for the polar CoA moiety and the hydrophobic fatty acid moiety to take separate pathways through the peroxisomal membrane, i.e. fatty acids potentially interact with the TMDs and/or flip-flop in the membrane whereas CoA may be transported through a hydrophilic channel in the TMDs or alternatively could be imported by another transporter, such as a member of the mitochondrial carrier family [49]. However, the lipid-linked oligosaccharide (LLO) flippase, PglK from *Campylobacter jejuni* transports amphipathic substrates without substrate cleavage. Recently, the determination of high resolution crystal structures for PglK coupled with biochemical assays and mutagenesis have provided evidence for a new model for substrate binding and translocation [50] (Figure 3A).

Three crystal structures were obtained for the PglK E510Q mutant: two representing the inward-facing, apo-state with widely separated NBDs (proposed to be transient in physiological situations, owing to the affinities of ABC transporters NBDs for nucleotides and the millimolar concentrations of ADP and ATP within cells) and a novel outward-occluded, ADP-bound conformation [50]. An outward-facing, ATP-bound state was modelled on Sav1866 [46]. In the proposed flipping model (Figure 3A), LLO approaches the outward-occluded ADP-bound transporter from the membrane where the polyprenyl tail interacts with a short α-helix (designated the external helix), situated parallel to the plane of the membrane at the periplasmic membrane boundary. Following ATP/ADP exchange, the pyrophosphate moiety is transferred into the outward-facing cavity, forming electrostatic interactions with a band of arginine residues. The oligosaccharide chain then follows, apparently without making specific contacts with the cavity. ATP hydrolysis brings about loosening of the contacts between the NBDs which is transmitted to the TMDs, causing pressure to be exerted on the substrate and permitting diffusion of the pyrophosphate and oligosaccharide moieties out of the translocation pathway. The polyprenyl tail is then released from the outward-occluded transporter, completing LLO flipping to the outside leaflet of the periplasmic membrane. An alternating access-type mechanism switching between an inward-open state with widely-separated NBDs to a classical outward-open, ATP-bound state was rejected for several reasons. Not only was the nucleotide-free state considered unphysiological, or at best, transient, but the large inward-facing cavity in the apo-conformation was also discounted as a potential binding site via mutagenesis experiments. Furthermore, the domain-swapped architecture of the transporter dictates that a transition between inward-open and outward-open states would result in a steric clash, trapping the LLO substrate and preventing release [50].

The generality of this model has been questioned since it is markedly different from the alternating access model proposed for the well-characterized *E. coli* lipid flippase, MsbA, in which the entire lipid is proposed to enter the inward-facing cavity [51–53]. Reconstituted MsbA exhibits lipid flippase activity towards a variety of membrane lipids *in vitro* but its physiological role is to flip lipid A from the inner to the outer leaflet of the cytoplasmic membrane [54]. The PglK mechanism requires a long, flexible lipid substrate and may therefore represent a distinct case where the nature of the substrate permits a different transport mechanism. Yet another lipid flipping mechanism is represented by ATP-independent LLO scramblase proteins which do not belong to the ABC transporter family [51].

## A tentative transport mechanism for CTS based on TAP

Previously, we proposed a transport mechanism for CTS based on an alternating access model of the human transporter associated with antigen processing (TAP) (Figure 3B) [4]. TAP is a heterodimeric ABC protein composed of two half transporters, TAP1 and TAP2. In common with CTS, TAP has asymmetric ATPase sites: one site is formed by consensus residues and is competent in ATP hydrolysis, whereas the other, degenerate site contains non-consensus sequences in the Walker B and switch motifs which reduce ATPase activity and substitutions in the signature motif, which decrease NBD dimer stability [55]. Although the degenerate site has residual hydrolytic activity it is not essential for transport. It is not yet known whether the non-canonical nucleotide binding site of CTS is competent in ATP hydrolysis, but it is dispensable for function *in vivo* [47,56]. Although the TAP model dictates that the NBD dimer must be open at both ATPase sites to trigger the inward-facing conformation, substitutions in both signature motifs of CTS will contribute to weakening of the NBD contacts in the absence of hydrolysis at both sites [47,55]. Unlike the situation for PglK, where an alternating access model precludes substrate release, the movements associated with the transition between inward- and outward-facing conformations of CTS could potentially contribute to its ATP-dependent thioesterase activity by repositioning catalytic residues, thus facilitating substrate translocation. In this context, one might envisage an evolutionary route whereby an ancestral transporter acquired thioesterase activity via gain of mutations in residues which promote catalysis (for example by binding and polarizing the thioester carbonyl of the acyl-CoA).

## Conclusions and open questions

Whilst an alternating access/thioesterase model for asymmetric ABC transporters has many plausible features and is supported by experimental evidence for TAP, several questions arise when extending this model to CTS: (1) In the broad specificity *Arabidopsis* transporter, specific binding of the CoA moiety provides a potential explanation for how disparate CoA esters might be recognized. However, this then raises the question of how diverse free acids traverse the membrane, following thioesterase-catalysed cleavage. It is conceivable that fatty acids could flip-flop from the outer to the inner leaflet of the peroxisomal membrane but is it necessary to invoke an alternative translocation pathway for more polar molecules, such as IBA and OPDA? (2) The mammalian ABCD transporters exhibit greater specificity for the acyl moiety of the transported substrate: how can this be reconciled with a model in which the liberated fatty acid flip-flops in the membrane? Interestingly, ABCD proteins do not possess the equivalent of the external helices proposed to provide binding specificity for the hydrophobic substrate moiety in PglK. (3) Although both plant and yeast peroxisomal ABC transporters are catalytically as well as structurally asymmetrical, the mammalian transporters are homodimers with two consensus ATPase sites. How does this affects the transport cycle? The alternating access/thioesterase model, although speculative, offers a useful hypothesis on which to base further experimentation to address these and other interesting questions. However, it is clear that evolution has provided more than one solution to the requirement for transport of amphipathic lipids across membranes.

## Abbreviations

ACS: acyl-CoA synthetase
AAE: acyl activating enzyme
ABC: ATP binding cassette
ABCD: ABC subclass D protein
ALDP: adrenoleukodystrophy protein
ALDR: adrenoleukodystrophy related protein
CTS: comatose
FA: fatty acid
IBA: indole butyric acid
LCFA: long chain fatty acid
LLO: lipid-linked oligosaccharide
MCFA: medium chain fatty acid
NBD: nucleotide binding domain
OPDA: 12-oxophytodienoic acid
PMP70: 70 kDa peroxisomal membrane protein
TAP: transporter associated with antigen processing
TMD: transmembrane domain
VLCFA: very long chain fatty acid
